# Editorial: Molecular physiopathology of epithelial ovarian cancer: role of inflammation

**DOI:** 10.3389/fonc.2023.1357842

**Published:** 2024-01-05

**Authors:** Gitana Maria Aceto, Dong-Joo Cheon

**Affiliations:** ^1^ Department of Medical, Oral and Biotechnological Sciences, Univeristy of Studies G. d’Annunzio University of Chieti-Pescara, Chieti, Italy; ^2^ Department of Regenerative and Cancer Cell Biology, Albany Medical College, Albany, NY, United States

**Keywords:** epithelial ovarian cancer, inflammation, pyroptosis, serum lipid and lipoprotein levels, aging, lymphadenectomy, vagus nerve

Inflammation emerges as an important risk factor associated with epithelial ovarian cancer (EOC), the most common type of ovarian cancer. Increased inflammation and inflammatory mediators are also associated with poor prognosis and shorter survival in EOC patients. Inflammation can be caused by several factors such as repeated ovulation, peritoneal infection, endometriosis, obesity, or exposure to foreign particles (e.g., pollutants). A chronic inflammatory microenvironment is established by increased levels of reactive oxygen species (ROS), cytokines, chemokines, and growth factors produced by the ovaries, immune cells, tumor cells, and tumor stroma. This inflammatory microenvironment can cause DNA damage, activation of signaling pathways, epigenetic alterations, chromosomal aberrations, and the impaired innate and adaptive immune response to promote tumor cell initiation, progression, metastasis, and drug resistance (reviewed in Savant SS et al., Cancers (Basel). 2018 Jul 30;10(8):251). This Research Topic was proposed to collect new studies that address how chronic inflammation, caused by individual characteristics (e.g., genetics and hormone levels) and environmental factors (e.g., infections and pollutants), can promote EOC initiation, progression, immunosuppression, and therapy resistance. Five articles in this Research Topic provide some insights on how inflammation could affect EOC progression.

Pyroptosis is a type of inflammatory-based cell death in response to various molecular patterns associated with damage and pathogens. The process activates a characteristic inflammasome and the executing proteins belong to the gasdermine family. Canonical pyroptosis is characterized by influx of sodium with water, swelling of cells, and osmotic cellular lysis, which results in release of pro-inflammatory intracellular content. Pyroptosis function is to destroy the intracellular pathogenic niche by inducing pro-inflammatory responses in neighboring cells. The tumor’s immune microenvironment can be influenced by pyroptosis in such a way as to favor the anti-tumor immune response. Liu et al. summarizes the current research of pyroptosis in ovarian cancer and discusses the promises and the challenges of targeting pyroptosis for ovarian cancer treatment.

The changes in lipids and lipoproteins that occur during inflammation are part of the innate immune response and are likely to play an important role in protecting the host. Huang et al. investigated how serum lipid levels affect EOC patients receiving bevacizumab treatment. They discovered a substantial increase in total cholesterol, triglycerides, and free fatty acids in those patients. They also developed a model predicting progression-free survival of EOC patients receiving bevacizumab treatment by including these serum lipid levels, which can be helpful in selecting a better treatment plan for patients.

Changes in lipid metabolism and metabolites are commonly observed in aging and may alter the innate immune response. Aging is accompanied by low-grade chronic inflammation, termed “inflammaging” characterized by increased levels of pro-inflammatory cytokines. This low-grade chronic inflammation represents a significant risk factor for a broad range of age-related diseases including cancer. Zhou et al. analyzed the risk factors of elderly women with EOC and generated a nomogram model that can accurately assess the overall survival of women older than 70 years with EOC at the time of the first treatment. This model can be helpful for individualized clinical treatment in elderly patients.

Systemic inflammatory status may be indicative of the prognosis of EOC patients, but its value is currently limited. Lymph node dissection in the EOC at an early stage for pathological evaluation may allow accurate staging of tumors in anticipation of disease progression and patient clinical management. However, in the early stage of EOC, it is unclear how to perform lymphadenectomy to avoid stage migration and achieve reliable targeted excision. Li et al. discovered that excision with appropriate numbers of lymph node draining the affected ovary may be more reasonable than traditional sentinel lymph node resection and systematic lymphadenectomy.

Chronic inflammation has been correlated with decreased vagus nerve activation. Indeed, there is increasing scientific evidence on the role of the vagus nerve in improving ‘vagal tone’ for the reduction of stress and inflammation. Cherifi et al. reports that low vagus nerve activity, measured by low heart rate variability, is associated with worse overall survival in ovarian cancer patients, suggesting new clinical applications of therapeutic neuromodulation, both pharmacological and non-pharmacological.

More studies are needed to get a more complete understanding of the role of inflammation in EOC initiation and progression. Especially we need more studies that help elucidate the role of the DNA damage repair systems in the ovarian and fallopian tube epithelium in relation to signaling pathways of epithelial renewal and immune system surveillance. Understanding these mechanisms would be important for developing novel therapeutic strategies that target inflammatory mediators to reduce ovarian cancer risk.

## Author contributions

GA: Writing – original draft, Writing – review & editing. D-JC: Writing – original draft, Writing – review & editing.

